# Examining internet use for health information seeking and influencing factors among undergraduate health science students in Southwest Ethiopia

**DOI:** 10.1016/j.heliyon.2024.e41545

**Published:** 2024-12-27

**Authors:** Alex Ayenew Chereka, Fikadu Wake Butta, Addisalem Workie Demsash, Sisay Yitayh Kassie, Adamu Ambachew Shibabaw, Abiy Tasew Dubale, Geleta Nenko Dube, Mekonnen Kenate Hunde, Wubalem Dilie Limeneh, Amare Dagnew Fentahun, Milkias Dugassa Emanu, Mekashaw Tareke Abebe, Gemeda Wakgari Kitil

**Affiliations:** a Department of Health Informatics, College of Health Sciences, Mattu University, Mattu, Ethiopia; b Department of Lifelong Learning & Community Development, College of Education and Behavioral Science, Mattu University, Mattu, Ethiopia; c Department of Tourism Management, College of Business and Economics, Injibara University, Ethiopia; d Department of Public Health, College of Medicine and Health Sciences, Injibara University, Ethiopia; e Department of Nursing, College of Health Sciences, Mattu University, Mattu, Ethiopia; f Department of Accounting and Finance, College of Business and Economics, University of Gondar, Gondar, Ethiopia; g Department of Midwifery, College of Health Sciences, Mattu University, Mattu, Ethiopia

**Keywords:** Internet use, Health information, Student, Utilization, Ethiopia

## Abstract

**Background:**

The Internet has become a pivotal resource for accessing health information globally, offering unprecedented convenience and breadth of resources. This cross-sectional study examines the implications of Internet use for health information seeking and the influencing factors among undergraduate health science students in Southwest Ethiopia.

**Methods:**

An institutional-based cross-sectional study was conducted from November 10 to December 10, 2023. The study enrolled 423 students. Data were collected using a pretested and structured self-administered questionnaire. Data entry and analysis were performed using EpiData V4.6 and STATA V17, respectively. Bi-variable and multivariable logistic regression analyses were conducted to identify factors associated with the outcome variable. Statistical significance was determined using a P-value of less than 0.05.

**Result:**

A total of 423 respondents participated in this study, resulting in a 100 % response rate. Among them, 67.4 % (95 % CI: 63.0%–72.0 %) reported using the Internet for health purposes. Factors significantly associated with this usage included being a third-year student or above (AOR = 2.33, 95 % CI: 1.36–3.99), owning a smartphone (AOR = 2.41, 95 % CI: 1.14–5.10), having good awareness (AOR = 2.81, 95 % CI: 1.55–5.09), holding a favorable attitude (AOR = 9.58, 95 % CI: 5.10–18.00), and possessing adequate e-health literacy (AOR = 5.25, 95 % CI: 2.89–9.46).

**Conclusion:**

This study underscores the internet's primary role in accessing information on diseases, treatments, healthy lifestyles, and prevention. Key factors such as e-health literacy, attitude, awareness, smartphone usage, and study timing significantly influence this trend. Understanding these factors is vital for optimizing internet resources for health information dissemination.

## Highlights

• **High Prevalence of Internet Use:** The study found that 67.4 % of undergraduate health science students in Southwest Ethiopia use the Internet for health information, indicating a high reliance on digital resources for health-related inquiries.
• **Comprehensive Study Design:** Conducted from November 10 to December 10, 2023, this institutional-based cross-sectional study achieved a remarkable 100 % response rate from 423 participating students, ensuring robust and representative findings.
• **Key Influencing Factors Identified:** Significant factors influencing internet use for health information included:
• **Academic Level:** Third-year students or above were more likely to use the internet for health purposes (AOR = 2.33, 95 % CI: 1.36–3.99).
• **Technology Access:** Ownership of a smartphone significantly increased the likelihood of using the internet for health information (AOR = 2.41, 95 % CI: 1.14–5.10).
• **Awareness and Attitude:** Students with good awareness (AOR = 2.81, 95 % CI: 1.55–5.09) and a favorable attitude towards internet use (AOR = 9.58, 95 % CI: 5.10–18.00) were more likely to seek health information online.
• **E-Health Literacy:** Adequate e-health literacy was strongly associated with increased internet use for health information (AOR = 5.25, 95 % CI: 2.89–9.46).
• **Robust Statistical Analysis:** Data were meticulously collected using a pretested and structured self-administered questionnaire, with data entry and analysis performed using EpiData V4.6 and STATA V17. Bi-variable and multivariable logistic regression analyses were employed to identify significant factors, ensuring the reliability of the findings.
• **Importance of E-Health Literacy:** The study underscores the critical role of e-health literacy in enabling students to effectively use internet resources for accessing health information, emphasizing the need for educational interventions to improve these skills.
• **Challenges and Barriers:** The research highlights common barriers faced by students, such as issues with internet connectivity and lack of digital literacy, which impede access to reliable health information online.
• **Preferred Information Sources:** The internet is primarily used by students to find information on diseases, treatments, healthy lifestyles, and prevention, reflecting their reliance on digital platforms for comprehensive health knowledge.
• **Educational Implications:** The findings suggest a need to integrate digital literacy and critical evaluation of online health information into the health sciences curriculum. This would better equip students with the skills necessary to navigate and assess the vast array of health information available online.
• **Recommendationsfor Improvement:** The study recommends targeted interventions to enhance access to reliable health information and improve digital literacy, awareness, and attitudes towards internet use among health science students. These steps are vital for optimizing the benefits of internet resources for health information dissemination.

## 1 Background

Globally, the Internet has become a crucial source of health-related information for individuals [1,2]. Access to accurate and reliable health information is vital for individuals to make informed decisions about their well-being [[2], [3], [4]]. With rapid technological advancements, the Internet has increasingly emerged as a primary platform for seeking health information [5,6]. This shift holds particular significance for university students, including those in health sciences. On one hand, students turn to the Internet for personal health management, utilizing e-health strategies and online self-care resources to manage their health. On the other hand, health science students rely on the Internet to support their professional development, accessing specialized knowledge and resources critical for their training as future healthcare providers [5,6]. Since the 1990s, an increasing number of people have turned to the Internet as a primary means of obtaining health-related information [7].

The Internet holds significant potential for advancing preventive health efforts, particularly when aimed at health science students, who represent a primary user group of online resources [8]. As a recent advancement in technology, the Internet facilitates global access to up-to-date health information [9]. Various platforms, including websites, forums, blogs, and social networks, are extensively employed for health promotion, collectively enhancing the quality of healthcare information through innovations in telemedicine and evidence-based practice [8,10].

The exponential growth of telemedicine and evidence-based medicine has significantly reshaped medical practice through the widespread influence of the Internet [11]. Furthermore, artificial intelligence (AI) has emerged as a critical field within healthcare, expanding capabilities in data analysis, personalized care, and clinical decision support [12,13]. AI applications in health information processing and telemedicine are revolutionizing healthcare delivery, allowing both students and professionals access to accurate, real-time insights that inform preventive and therapeutic practices [14,15]. This integration of AI and internet-based resources is transforming healthcare education and practice, equipping health science students with the ability to access, critically evaluate, and effectively apply reliable health information [16,17].

Across the globe, people are increasingly turning to the Internet to discover and endorse health-related information [18,19]. The global surge in internet users has been remarkable, with statistics indicating a substantial increase. From 2013 to 2023, the number of worldwide internet users expanded from 2.53 billion to 5.16 billion, and in Africa, the figures rose from 162.62 million to 580.27 million during the same period [20]. This rapid increase in internet access shows that more individuals are relying on online platforms for health information [21].

Despite the number of internet users dramatically increasing and the fact that the internet is a valuable resource for health information for an individual, the majority of previous research suggested that people had a low culture of using the internet to obtain health-related information [9,22]. Recent data from 2022 highlighted that out of 5.4 billion internet users globally, only 50 million people utilized the internet for accessing health-related information. Which is a very small proportion, This usage stands in stark contrast to the overall magnitude of internet users [23,24].

The utilization of the internet for obtaining health information varies across regions, revealing distinct patterns. In the UK, 97 % of individuals sought health information online [25], while in Jeddah, the figure was 92.7 % [26]. In Malaysia, 80 % of the population used the Internet for health-related information [27], whereas in Ghana, the percentage was 53 % [28]. In Addis Ababa, Ethiopia, the utilization rate was 59.1 % [29], while Kuwait registered 62.9 % [30], and Gondar, Ethiopia, reported 47.4 % [31]. These statistics suggest that the inclination to use the internet for health information is more prevalent in developed countries, as evidenced by the high percentages in the UK and Jeddah. Conversely, the figures in developing countries such as Ghana, Addis Ababa, and Gondar indicate a comparatively lower adoption of internet usage for health information [25,26].

Accessing health information through the Internet is essential for various professions and plays a significant role in enhancing a country's health system. Investigating how undergraduate health science students at Mattu University utilize the Internet for health information is crucial for several reasons. Firstly, this examination helps identify gaps in access to reliable health information, which can lead to targeted interventions that address specific challenges faced by students. Secondly, the findings can inform the development of health education programs that effectively use online platforms to disseminate accurate and relevant health information. Furthermore, this research contributes valuable insights to the field of health communication by illustrating how the Internet shapes health-seeking behaviors among undergraduate health science students. Overall, the primary aim of this study is to explore Internet use for health information seeking and the factors that influence this behavior among health science students in the Oromia region of Southwest Ethiopia. By understanding these dynamics, the study can provide recommendations to improve health information access and education for this key demographic.

## 2 Methods and materials

### 2.1 Study period, design, and area

From November 10 to December 10, 2023, an institution-based cross-sectional study was conducted at Mattu University Ethiopia. Mattu University, established in 2011 G C., is a prominent higher education institution in Ethiopia, classified as a third-generation university. Situated in Mattu town, the capital of the Ilu Aba Bora administrative zone in the Oromia Regional State, it is approximately 600 km away from the capital city, Addis Ababa.

Comprising two campuses and five colleges, Health Science College includes seven departments: Pharmacy, Public Health Officer, Health Informatics, Nursing, Midwifery, Psychiatry, and Medical Laboratory. This study aims to examine internet use for health information seeking and the influencing factors among undergraduate health science students at Mattu University in Southwest Ethiopia. The target population consists of health science students across these various departments. Health information was defined based on specific criteria, which included the types of health topics participants frequently searched for online, such as maternal health, child health, chronic diseases, mental health, nutrition, and preventive care. Participants identified their preferred online sources for health information, including academic websites, government health portals, social media, and peer-reviewed journals. Additionally, students reported the frequency with which they utilized the internet to seek health information, categorized as daily, weekly, monthly, or rarely. They were also asked to rate the credibility of the sources they used, which helped gauge their critical assessment of the information accessed. Data was collected using a structured questionnaire administered through face-to-face interviews.

### 2.2 Source and study populations

The source and study population for this research encompassed all undergraduate health science students at Mattu University. Inclusively, all undergraduate health science students available during the designated study period were considered participants. Nevertheless, exclusion criteria were applied to those enrolled in summer, post-basic, and weekend programs, as well as those unavailable during the data collection period due to reasons such as illness, family obligations, and other factors. This ensured a comprehensive representation of the target population while accounting for specific circumstances that might affect participation.

### 2.3 Sample size determination and sampling procedure

The total sample size was determined using the single population proportion formula: n = [(Zα/2)^2^P (1-P)]/d^2^). Since no previous studies had been conducted on the use of the Internet for health information among undergraduate health science students, a prevalence of 50 % was assumed. Considering a 95 % confidence interval (Zα/2 = 1.96), a margin of error (d) 5 %, and a 10 % non-response rate, the final sample size was calculated to be 423. A stratified sampling method was employed, followed by random sampling techniques, to recruit study participants. The sampling procedure involved proportionate stratified sampling based on the distribution of students across various departments within the health science college, which comprises seven departments: Midwifery (154 students), Health Informatics (125), Psychiatry (130), Medical Laboratory (126), Nursing (160), Health Officer (140), and Pharmacy (172), totaling a student population of 1007. To determine the sample size for each department, we calculated the proportion of students in each department relative to the total student population. The formula for proportionate sampling was applied as follows:

$$\text{Sample}\;\text{Size}\;\text{for}\;\text{Department}=\frac{\left(\text{Total}\;\text{Number}\;\text{of}\;\text{Students}\;\text{in}\;\text{Department}\right)\times \text{Total}\;\text{Sample}\;\text{Size}}{\text{Total}\;\text{Students}}\text{.}$$

Using this approach, we estimated the number of participants to recruit from each department: Midwifery (64), Health Informatics (53), Psychiatry (54), Medical Laboratory (52), Nursing (67), Health Officer (59), and Pharmacy (74).

### 2.4 Data collection tool and procedure

To ensure the integrity and dependability of the collected data, a pretested and structured self-administered questionnaire was employed during the data collection period. Recognizing the significance of the internet as a crucial resource for health information, the questionnaire was meticulously developed by drawing upon and modifying insights from various studies that focused on the utilization of the internet for health information-seeking. This meticulous approach aimed to enhance the tool's reliability and relevance to the specific context of health science students at Mattu University [19,29,[32], [33], [34], [35], [36]]. There were two supervisors and four data collectors involved in the data collection process. 45 questions in all, divided into five sections: information source, individual characteristics, students' usage of the Internet for health, students' sociodemographic variables, and organizational aspects. A pretest was conducted among 22 undergraduate health science students (5 % of the total sample size) at Jimma University, which was similar to our study setting. Based on the results of the pretest, the accuracy, consistency, and quality of the questionnaire were examined in detail. Expert opinion was used to assess the questionnaire's content validity, and Cronbach alpha was used to calculate the reliability of the results (the overall Cronbach alpha = 0.86).

### 2.5 Measurements

The use of the Internet to find health information was one of the study's dependent variables. A single-item question titled "Have you ever used the Internet to look for health or medical information?" was used to measure the outcome variables. The response was coded as (1 = 'Yes' if participants use the Internet for health purposes and 0 = 'No’ if participants do not use the Internet for health purposes) [29,37,38].

**Awareness** refers to the respondents’ knowledge and understanding of the availability, reliability, and potential benefits of using the internet for health information

**e-Health literacy:** It was measured by eight closed-ended Likert scale questions in which ratings were made on one to five scales where; 1 = strongly disagree, 2 = disagree, 3 = neutral, 4 = agree, and 5 = strongly agree. Those who achieved a mean score or higher on the e-health literacy scale were deemed to have a good level of proficiency in using the internet for health-related information. When it came to using the Internet for health information, respondents with scores below the mean were deemed to have low e-health literacy [29,39].

**Awareness of using the Internet for health information** was assessed using four closed-ended Likert scale questions, with responses ranging from "1 = strongly disagree" to "5 = strongly agree". Respondents achieving a mean score or higher were considered to have an excellent awareness of using the Internet for health-related research. Conversely, those scoring below the mean were classified as having low awareness of utilizing the Internet for health information [28].

**Attitude to use the Internet for health information:** the level of attitude was measured by fourteen closed-ended Likert scale questions in which ratings were made on one to five scales where; 1 = strongly disagree, 2 = disagree, 3 = neutral, 4 = agree, and 5 = strongly agree. Respondents who scored with the mean score and above were considered a favorable attitude toward using the internet for health information. Whereas, respondents who scored below the mean were considered an unfavorable attitude to use the Internet for health information [28].

**Good awareness:** refers to the level of knowledge that health science students have about using the internet for seeking health information. It was measured by a set of knowledge-based questions in the questionnaire, assessing the students' familiarity with reliable online health resources, search strategies, and evaluation of information credibility. Participants were classified as having good awareness if they answered correctly on 70 % or more of these questions.

**A favorable attitude** refers to the positive perception and openness of health science students toward using the Internet as a source of health information. This was measured through a series of Likert-scale statements in the questionnaire, with responses ranging from 1 (strongly disagree) to 5 (strongly agree). Students who scored 70 % or higher in favor of positive statements—such as trust in online sources and ease of use—were considered to have a favorable attitude toward internet use for health information.

### 2.6 Data processing and analysis

To ensure the accuracy and consistency of the data, we first coded and cleaned it. The data was then imported into EpiData version 4.6 and exported to STATA version 17 for statistical analysis. Frequency tables were used to present summary data for sociodemographic variables. To control for confounding factors, a bivariable logistic regression analysis was initially performed. Independent variables with a P-value less than 0.2 in the bivariable analysis were then included in the multivariable logistic regression analysis.

Multicollinearity among the independent variables was assessed, and the appropriateness of the model was evaluated using the Hosmer-Lemeshow test. Adjusted odds ratios (AOR) with 95 % confidence intervals (CIs) were calculated to assess the strength of associations, and statistical significance was determined at a p-value of <0.05.

Model fitness was further checked using the Hosmer-Lemeshow test (χ^2^/DF = 4.81; RMSEA = 0.05; CFI = 0.95; TLI = 0.93). A multicollinearity test was also conducted, and all variables had variance inflation factors (VIFs) between 1.0 and 1.7, which are within acceptable limits.

## 3 Result

### 3.1 Socio-demographic characteristics

Mattu University's undergraduate health science students received a total of 423 questionnaires. A 100 % completion rate was attained for all questionnaires. Of the total respondents, 230 (54.4 %) of males, more than half of the respondents 231(54.6 %) were under 20–24 years old, most of the respondents 367(86.7 %) were Smartphone holders and around two-thirds, 275 (65.5 %) of respondents came from a rural place (Table 1).

**Table 1:** Socio-demographic characteristics of undergraduate health science students at Mattu University, Southwest, Ethiopia, 2023(n = 423).

Variables (n = 423)	Frequency (n)	Percentage %
Sex of the respondent		
Male	230	54.4
Female	193	45.6
Age category in years		
<20	74	17.5
20-24	231	54.6
25 and above	118	27.9
Years of study		
Second year	157	37.1
Third year	133	31.5
Fourth-year	99	23.4
Fifth year	34	8.0
Religions of the respondent		
Christian orthodox	173	40.9
Muslim	102	24.1
Protestant	103	24.3
Others	45	10.6
Departments of the respondent		
Public health officer	50	11.8
Nurse	90	21.3
Medical laboratory	30	7.1
Midwifery	88	20.8
Health informatics	47	11.1
Pharmacy	82	19.4
Psychiatry	36	8.5
Types of mobile phone		
Smart	367	86.7
Basic	56	13.3
Residence of the respondent		
Urban	148	35.0
Rural	275	65.5

### 3.2 Internet access and use by students

Based on respondents' internet access results showed that 100 % of respondents accessed the internet, around two-thirds 270 (63.8 %) of respondents were one to five years of experience, 360 (85.1 %) of respondents used the internet daily, around half 189 (44.7 %) of respondents used mobile data network and more than half 230 (54.4 %) of respondents primarily access their internet at home (Table 2).

**Table 2:** Internet access and used by undergraduate health sciences students at Mattu University, Southwest, Ethiopia, 2023(n = 423).

Variables (n = 423)	Frequency (n)	Percentage %
**Use of Internet**		
Yes	423	100
No	0	0
**Years of Internet use**		
<1	74	17.5
1-5	270	63.8
>5	79	18.7
**Use of the Internet every day?**		
Yes	360	85.1
No	63	14.9
**Hours of daily Internet use**		
1_3	173	40.9
4_7	102	24.1
8_10	103	24.3
>10	45	10.6
**Barriers to Internet use**		
Unreliable and slow connection	161	38.1
High cost of internet and device	120	28.3
Unreliable power supply	113	26.7
Viruses and malware	18	4.3
No challenge	11	2.6
**Types of Internet use**		
Mobile data	189	44.7
Campus WIFI	107	25.3
From both	127	30.0
**Place of primary Internet access**		
Campus and lab WIFI	107	25.3
Halls and hostels	86	20.3
Home	230	54.4

### 3.3 Individual characteristics

Based on our findings, from the total study participants, 308 (72.8 %) of respondents had good awareness of using the Internet for health purposes, 327 (77.3 %) of respondents had a favorable attitude to use of the Internet for health information, more than half 281 (66.4 %) of students were motivated to use the internet for health purpose, most 340 (80.4 %) of respondents were e-health literate, and more than half 221(52.2 %) of the respondents said internet is useful for accessing health information.

### 3.4 Use of the internet for health purposes

Of the total internet users, 344 (81.3 %) respondents were social media users, more than two-thirds 290 (68.6 %) respondents were online mobile app users, around three-fourths 312 (73.7 %) of respondents were e-health literate, and around two-thirds, 281 (66.4 %) of respondents were general search engine users (Table 3). Moreover, the proportion using the Internet for health information among undergraduate students was 67.4 %(95 % CI: 63.0–72.0).

**Table 3:** Use of the Internet for health purposes among undergraduate health sciences students at Mattu University, Southwest, Ethiopia, 2023(n = 423).

Variables (n = 423)	Frequency (n)	Percentage %
Using social media for health information		
Yes	344	81.3
No	79	18.7
Using mobile-based online apps for health information		
Yes	290	68.6
No	133	31.4
Using e-health or hospital database		
Yes	312	73.7
No	111	26.3
Using email for health information		
Yes	294	69.5
No	129	30.5
Using virtual channels for health information		
Yes	190	44.9
No	233	55.1
Using a general search engine health information		
Yes	281	66.4
No	142	33.6

### 3.5 Factors associated with internet use for health information

From the total variables entered into the bi-variable logistic regression model, sex, age, types of mobile phone, attitude, motivation, e-health literacy, awareness, and years of study were factors associated with the use of the internet for health information in the bi-variable analysis at *P*-value less than 0.2. Consequently, those variables were subjected to the multivariable logistic regression analysis to control potential confounders.

In the multivariable logistic regression, third years and above students [AOR = 2.33, 95%CI(1.36–3.99)], Smartphone holders [AOR = 2.41, 95%CI(1.14–5.10)], good awareness [AOR = 2.81, 95%CI(1.55–5.09)], favorable attitude [AOR = 9.58, 95%CI(5.10–18.00)], and adequate e-health literacy [AOR = 5.25, 95%CI(2.89–9.46)] were significantly associated with overall use of the internet for health information among undergraduate health sciences students at Mattu university, at *P*-value less than 0.05 (Table 4).

**Table 4:** Factors associated with internet use for health information among undergraduate health science students at Mattu University, Southwest, Ethiopia, 2023(n = 423).

Variables (n = 423)	Internet use for health information		COR(95%CI)	AOR (95 %)
	Yes	No		
Age category in years				
<20	47(11.1 %)	27(6.4 %)	1	1
20-24	154(36.4 %)	77(18.2 %)	1.15(0.67–1.99)	1.94(0.95–4.00)
25 and above	84(19.9 %)	34(8.0 %)	1.42(0.77–2.64)	2.18(0.97–5.10)
Sex				
Female	151(35.7 %)	79(18.7 %)	1	1
Male	134(31.7 %)	59(13.9 %)	1.19 (0.67–1.99)	1.09(0.64–1.88)
Years of study				
Second below	117(27.7 %)	67(15.8 %)	1	1
Third and above	168(39.7 %)	71(16.8 %)	1.36(1.01–2.04)	2.33(1.36–3.99)∗
Types of devices				
Basic phone	21(5.0 %)	35(8.3 %)	1	1
Smartphone	264(62.4 %)	103(24.3 %)	4.27(2.38–7.68)	2.41(1.14–5.10)∗
Awareness				
Poor	46(10.9 %)	69(16.3 %)	1	1
Good	239(56.5 %)	69(16.3 %)	5.20(3.28–8.22)	2.81(1.55–5.09)∗
Motivation				
Poor	86(20.3 %)	56(13.2 %)	1	1
Good	199(47.1 %)	82(19.4 %)	1.58(1.04–2.41)	1.38(0.79–2.39)
Attitude				
Unfavorable	26(6.1 %)	70(16.6 %)	1	1
Favorable	259(61.2 %)	68(16.1 %)	10.26(6.08–17.3)	9.58(5.10–18.0)∗
e-health Literacy				
Inadequate	44(10.4 %)	66(15.6 %)	1	1
Adequate	241(57.0 %)	72(17.0 %)	5.02(3.16–7.98)	5.25(2.89–9.46)∗

## 4 Discussion

Examining Internet use for health information seeking among undergraduate health science students in Southwest Ethiopia is crucial for improving self-care and supporting academic training. By accessing online resources, students can make informed health decisions, leading to healthier behaviors and better overall wellness. This is especially important in resource-limited settings [40,41]. The Internet provides students with valuable scholarly articles, research studies, and educational materials, enhancing their learning experiences and keeping them updated on current health trends and evidence-based practices essential for their future careers [42].

The findings of this study revealed that a significant proportion of undergraduate health science students at Mattu University in Southwest Ethiopia use the Internet to access health information. Students should increasingly utilize the Internet as a valuable resource for sharing health information and preparing for their future employment [43]. While there are several methods for obtaining health information online, our study focused specifically on undergraduate health sciences students. Overall, the study showed that 100 % of the respondents had access to the Internet; among them, 285 (67.4 %) (95 % CI; 63.0–72.0 %) reported using the Internet for health information.

This finding was in line with the study conducted on Ghana University students (67.7 %) [35]. Additionally, this finding was higher than the studies conducted in Ghana adolescents (53 %) [28], Addis Ababa (59.1 %) [29], Kuwait (62.9) [30], and Gondar Ethiopia (47.4 %) [31]. This disparity might be the study population; most of the study population we assessed was general university or college students, not specific health science students. However, this study was focused on specifically undergraduate health science students at the university. Due to this, health science students are more likely to use the internet for health-related information.

However, this finding was less than the studies done UK (97 %) [25], Jeddah (92.7 %) [26], and Malaysia (80 %) [27]. This variation could be the study setting. When have seen the Ethiopian internet penetration, was less than the African average internet penetration [29,44,45]. According to the 2017 World Bank report, Ethiopia has very low internet penetration (20 %) [46]. This might be a significant difference between the studies.

This study has five significant variables; such as respondents' e-health literacy level, attitude, awareness, years of study, and Smartphone device holders. According to the multivariable regression analysis result, the odds of respondents who had an adequate level of e-health literacy to use the internet for health information were 5.25 times more likely to use the Internet for health information than that of respondents who had an inadequate level of e-health literacy. This finding was consistent with the study done UK [25], North Florida [47], Israel [48], and Ethiopia [29]. This implied that, In comparison to people with poor e-health literacy, those with adequate e-health literacy are more likely to utilize the internet for health information and have higher information demands.

The odds of undergraduate health science students who were in third years and above were 2.33 times more likely to use the internet for health information than that of respondents in the second year and below years of study. This showed that the year level of study increased among respondents the knowledge, attitude, and awareness also increased to use the internet for health information. I.e. the respondents understand the advantages of the internet for health purposes. This study was supported by the studies done in Botswana [49], Kuwait [30], Japan [50], Gondar Ethiopia (47.4 %) [31], and Addis Ababa Ethiopia [29].

The odds of respondents who had Smartphone access were 2.41 times more likely to use the internet for health information than that of the respondents who had basic phones. This could be if respondents have Smartphones they could simply use important applications that help to access health-related information. The other possible reason for this finding could be the availability of smartphones could enable to use of social media platforms, email, channels, and other general search engines that help to access health and health-related information. This study was consistent with the study conducted in Ethiopia [29].

In addition, a favorable attitude and good awareness were significant factors for the use of the Internet for health information among respondents. As a result, the awareness and attitudes of respondents increase and the use of the internet for health information also increases. If the respondents know the advantages of the internet for health information, they frequently use the internet for health information. However, most of the previous studies did not assess the attitude and awareness of the respondents. Due to this, we, the authors, cannot support this study.

### 4.1 Strengths and limitations of the study

This study addresses an important and current issue, as the use of the internet for health information is becoming increasingly prevalent worldwide. Understanding the factors associated with this behavior among undergraduate students can provide valuable insights for healthcare professionals and educators. However, the study focused specifically on undergraduate health science students in Southwest Ethiopia. Therefore, the findings may not be generalizable to other populations or regions. It would be beneficial to conduct similar studies in different contexts to obtain a more comprehensive understanding of the topic.

## 5 Conclusion

The findings indicate that the primary purpose of internet use among students was to seek information about diseases, treatment options, healthy lifestyles, and preventive measures. Several factors were identified as significant in the use of the Internet for health information, including a sufficient level of e-health literacy, a favorable attitude, good awareness, smartphone ownership, and being in the third year or above of study. These findings underscore the importance of promoting internet access and enhancing digital health literacy among undergraduate health science students in Southwest Ethiopia.

## CRediT authorship contribution statement

**Alex Ayenew Chereka:** Writing – review & editing, Writing – original draft, Visualization, Validation, Supervision, Software, Resources, Project administration, Methodology, Investigation, Funding acquisition, Formal analysis, Data curation, Conceptualization. **Fikadu Wake Butta:** Writing – original draft, Formal analysis, Data curation, Conceptualization. **Addisalem Workie Demsash:** Writing – review & editing, Writing – original draft, Visualization, Validation, Supervision, Formal analysis, Data curation, Conceptualization. **Sisay Yitayh Kassie:** Writing – review & editing, Writing – original draft, Visualization, Validation, Conceptualization. **Adamu Ambachew Shibabaw:** Writing – review & editing, Writing – original draft, Visualization, Formal analysis, Conceptualization. **Abiy Tasew Dubale:** Writing – original draft, Visualization, Validation, Supervision, Formal analysis, Data curation, Conceptualization. **Geleta Nenko Dube:** Writing – review & editing, Writing – original draft, Visualization, Validation, Conceptualization. **Mekonnen Kenate Hunde:** Writing – review & editing, Supervision, Methodology, Data curation, Conceptualization. **Wubalem Dilie Limeneh:** Writing – review & editing, Visualization, Methodology, Data curation. **Amare Dagnew Fentahun:** Writing – review & editing, Visualization, Validation, Supervision, Formal analysis, Data curation, Conceptualization. **Milkias Dugassa Emanu:** Visualization, Formal analysis, Conceptualization. **Mekashaw Tareke Abebe:** Writing – review & editing, Writing – original draft, Supervision, Data curation, Conceptualization. **Gemeda Wakgari Kitil:** Writing – review & editing, Writing – original draft, Visualization, Supervision, Software, Methodology, Formal analysis, Data curation, Conceptualization.

## Research ethics approval

Ethical clearance was obtained from the ethical review committees of Mattu University with the ethical reference number: RPG/299/2015 E C (RPG/299/2023 G C). Informed consent was obtained from each study participant after introducing the objective and benefits of the study. To keep the confidentiality of information provided by the study subjects, the data collection procedure was anonymous. The study was conducted as per the declaration of Helsinki.

## Consent for publication

Not applicable.

## Data availability

The data will be available at any time when requested by the corresponding author.

## Funding

There is no funding for this research.

## Declaration of competing interest

The authors declare the following financial interests/personal relationships which may be considered as potential competing interests:Reports a relationship with that includes:. Has patent pending to. If there are other authors, they declare that they have no known competing financial interests or personal relationships that could have appeared to influence the work reported in this paper.

## Abbreviations

**AOR**: Adjusted Odds Ratio
**CFI**: Comparative Fit Index
**CI**: Confidence Intervals
**COR**: Crude Odds Ratio
**FMOH**: Federal Ministry of Health
**SPSS**: Statistical Package for Social Science
**UK:**: United Kingdom
**VIF**: Variance Inflation Factor
**WHO**: World Health Organization
**WHZ**: Weight-for-height z-scores.
