# Supplementary material for: Examining internet use for health information seeking and influencing factors among undergraduate health science students in Southwest Ethiopia
Source: Heliyon. 2024 Dec 27;11(1):e41545. doi: 10.1016/j.heliyon.2024.e41545 (PMC11750529; doi:10.1016/j.heliyon.2024.e41545)
Supplement: Multimedia component 1 [file mmc1.docx]

## **Informed Consent and Certification Form**

This questionnaire is intended to assess Internet use for health information purposes and associated factors among undergraduate Health science students at Mattu University, Ethiopia.

You are kindly requested to be included in the study, which will have importance in improving the health service delivery system of the country. The interview will take about 15 minutes. No information concerning you as an individual will be passed to another individual or institution without your agreement. Your participation is voluntary, and you have the right not to participate fully or partially.

If you agree to be included in the study, I will start my questions by asking general identification points. Only honest answers would contribute to the improvement of health planning. The study has been approved by the Mattu University

May I continue?

Yes. No.

Having been well explained and informed of the intentions and benefits of the study, I am voluntary to participate in the study.

Respondent

Sign ____________

Date ______________

## **B: Questionnaire English Version**

| **Part: I Socio-demographic characteristics** | | | |
| --- | --- | --- | --- |
| S. No | Questions | Response | Remark |
| 101 | Age in years | (________________) |  |
| 102 | Sex | 1. Male  2. Female |  |
| 103 | Prior residence | 1. Urban  2. rural |  |
| 104 | Religion | 1. Orthodox  2. Muslim  3. Protestant  4. Catholic  5. Other specify ________________) |  |
| 105 | Ethnicity | 1. Amhara  2. Oromo  3. Tigray  4. Gurage  5. Other specify ­­­­­ |  |
| 106 | Educational level | 1. First year  2. Second year  3. Third year  4. Fourth year  5. Fifth year |  |
| 107 | Field of the study | 1.Health informatics  2. Nursing  3. Laboratory  4. Midwifery  5. Public health  6. Pharmacy  7. Psychiatry |  |
| 108 | Residence status | 1. In campus  2. Out of campus |  |
| 109 | Monthly Family income (In ETB) | ( ________________________) |  |

| **Part 2:** Internet access and used by undergraduate health sciences students at Mattu University | | |
| --- | --- | --- |
| 1 | Do you have internet access? | 1. Yes 2. 2. No |
| 2 | If you have an internet access how many years of use internet? | 1. <1year 2. 1-5 years 3. >5 years |
| 3 | Do you have use an internet every day? | 1. Yes 2. No |
| 4 | How much hours of do you have used internet every day? | 1. 1-3 hours  2. 4-7 hours  3. 8-10 hours  4. >10 hours |
| 5 | What types of internet access do you have? | 1. Mobile 2. Compass Wi-Fi 3. Others |
| 6 | Where are your primary places of internet access? | 1. 1. In the compass 2. 2. At home 3. 3. At hotels and private |
| 8 | What are the barriers/ factors to use an internet? | 1. Unreliable and slow connection, 2. High cost of internet and device, 3. Unreliable power supply, 4. Viruses and malware , 5. No challenge |

| **Part 2: Technological factor** | | | |
| --- | --- | --- | --- |
| 201 | Do you have one/more of the following devices?  (more than one answer is applicable) | 1. Smart Phone  2. Tablet computer  3. Lap top computer  4. Desktop computer  5. Others specify ­­­ -------- ) |  |
| 202 | Do you have access to the Internet via mobile data? | 1. Yes  2. No |  |
| 203 | Have you ever used the Internet for any purpose? | 1. Yes  2. No | If No 🡺301 |
| 204 | If yes to Q 203, what is your source of Internet?  (more than one answer is applicable) | 1.Mobile data  2.Institutional  3.Private |  |
| 205 | If yes, to Q 203, which device do you use for the Internet?  (more than one answer is applicable) | 1. Smart Phone  2. Tablet computer  3. Lap top computer  4. Desktop computer  5.Others specify______) |  |
| **Part 3: Use of the Internet for health Information** | | | |
| 301 | Have you ever used the Internet to look for health or medical information? | 1. Yes  2. No | If No 🡺303 |
| 302 | If yes, to Q 301, How many times in the last six month? | 1. Once  2. Twice  3. Three or more |  |
| 303 | Is there a specific website you especially like to go to for health or medical information? | 1. Yes  2. No | If No 🡺305 |
| 304 | Which medical information website do you mostly use?  (More than one answer is possible) | 1. WebMD  2. MEDLINE  3. Healthline  4. HealthFinder  5. Others Specify­­­­ ­­­­--------) |  |

| **Question from 305 to 310; please put a tick (√) mark under the alternatives given.** | | 1. Weekly | 2. Monthly | | 3. Rarely | 4. Never |  |
| --- | --- | --- | --- | --- | --- | --- | --- |
| 305 | How often did you interact with health care professionals before you started using the Internet for health information? |  |  | |  |  |  |
| 306 | How often did you participate in forums or self-groups aiming on health or sickness through the Internet? |  |  | |  |  |  |
| 307 | How often did you read health and illness information from the Internet? |  |  | |  |  |  |
| 308 | Do you use the Internet to find health information that can help you decide whether to consult a health care professional? |  |  | |  |  |  |
| 309 | Do you find health information by using the Internet earlier to a medical appointment? |  |  | |  |  |  |
| 310 | Do you find health information from the Internet after an appointment with health care professional? |  |  | |  |  |  |
| **Part 4: Did you use any of the following as a source for health information** | | | | | | | |
| 401 | Browsing online apps for smart phones and tablets? | | | 1. Yes  2. No | | |  |
| 402 | Using search engines e.g., Google and Yahoo? | | | 1. Yes  2. No | | |  |
| 403 | Websites like E-health website or Hospital websites? | | | 1. Yes  2. No | | |  |
| 404 | Using Virtual channels e.g., You tube | | | 1. Yes  2. No | | |  |
| 405 | Using email for health information | | | 1. Yes  2. No | | |  |
| 406 | Using social media e.g. Facebook for health information | | | 1. Yes  2. No | | |  |
| 407 | Based on the health information sourced from the Internet did you make cancel or change appointment with your health care provider? | | | 1. Yes  2. No | | |  |
| 408 | Did you discuss the health information sourced from the Internet with your health care professional (Doctor)? | | | 1. Yes  2. No | | |  |
| 409 | Did you change your medication based on the information gained from the Internet if any, without discussing it with your health care provider (Doctor)? | | | 1. Yes  2. No | | |  |
| 410 | Based on the health information gained using Internet did you request the diagnosis made by your health care provider (Doctor)? | | | 1. Yes  2. No | | |  |
| 411 | Based on the health information sourced using the Internet did you request the treatment given by your health care provider (Doctor)? | | | 1. Yes  2. No | | |  |
| 412 | Based on the health information gained from the Internet did you change your life style? | | | 1. Yes  2. No | | |  |
| 413 | Which type of health information you prefer most from the Internet? | | | 1. Text  2. Audio  3. Audio-vedio  4. Graphic | | |  |

| **Part 5: eHealth literacy**  I would like to ask your opinion and experience in using internet for health information.  For each statement, tick the answer to that you’re best view and experience. | | | | | | |  |
| --- | --- | --- | --- | --- | --- | --- | --- |
|  |  | Strongly disagree | Disagree | Neither agree nor Disagree | Agree | Strongly Agree | |
| 501 | I know which health information can be found on the internet |  |  |  |  |  | |
| 502 | I know where I can find useful1 health information on the internet |  |  |  |  |  | |
| 503 | I know how to find useful health information on the internet |  |  |  |  |  | |
| 504 | I know how I can use the Internet to answer my questions about health reply |  |  |  |  |  | |
| 505 | I know how I can use the health information found on the internet to help myself |  |  |  |  |  | |
| 506 | I have sufficient skills to get the health information that I find on it to value the internet |  |  |  |  |  | |
| 507 | I can distinguish between health information of high and low quality on the internet |  |  |  |  |  | |
| 508 | I feel confident about using information from the internet decisions about my health |  |  |  |  |  | |

**Thank you! For your cooperation!**
